# Environmental rRNA inventories miss over half of protistan diversity

**DOI:** 10.1186/1471-2180-8-222

**Published:** 2008-12-16

**Authors:** Sunok Jeon, John Bunge, Chesley Leslin, Thorsten Stoeck, Sunhee Hong, Slava S Epstein

**Affiliations:** 1 Department of Environmental Science, Kangwon National University, Kangwon-Do, Korea; 2 Dept. of Statistical Science, 301 Malott Hall, Cornell University, Ithaca, New York 14853, USA; 3 Department of Biology, Northeastern University, Boston, Massachusetts, 02115, USA; 4 School of Biology, University of Kaiserslautern, Erwin Schrödinger Str. 14, D-67663 Kaiserslautern, Germany; 5 Marine Science Center, Northeastern University, Nahant, Massachusetts, 01908, USA

## Abstract

**Background:**

The main tool to discover novel microbial eukaryotes is the rRNA approach. This approach has important biases, including PCR discrimination against certain rRNA gene species, which makes molecular inventories skewed relative to the source communities. The degree of this bias has not been quantified, and it remains unclear whether species missed from clone libraries could be recovered by increasing sequencing efforts, or whether they cannot be detected in principle. Here we attempt to discriminate between these possibilities by statistically analysing four protistan inventories obtained using different general eukaryotic PCR primers.

**Results:**

We show that each PCR primer set-specific clone library is not a sample from the community diversity but rather from a fraction of this diversity. Therefore, even sequencing such clone libraries to saturation would only recover that fraction, which, according to the parametric models, varies between 17 ± 4% to 49 ± 10%, depending on the set of primers. The pooled data is thus qualitatively richer than individual libraries, even if normalized to the same sequencing effort.

**Conclusion:**

The use of a single pair of primers leads to significant underestimation of the true community richness at all levels of taxonomic hierarchy. The majority of available protistan rRNA gene surveys likely sampled less than half of the target diversity, and might have completely missed the rest. The use of multiple PCR primers reduces this bias but does not necessarily eliminate it.

## Background

Over the past several years there has been a surge of studies applying the rRNA approach [1] to discover and inventory microbial eukaryotes in many environments [*cf.*, [2-4]]. These studies have documented an unprecedented diversity of novel protists at all levels of taxonomic hierarchy, and made an important contribution to the study of microeukaryotic richness, biogeography, and evolution. The cultivation-independent approach that employs cloning and sequencing of 18S rRNA gene fragments that are PCR-amplified from environmental genomic DNA will most likely continue to play a unique role in microbial discovery, especially since metagenomics approaches, so successful in bacterial and archaeal research [5,6], are less practical for microbial eukaryotes owing to their large genome size. It is therefore important to know if such approach misses some eukaryotes, and if so, how many, and how to minimize the bias.

It is well known that PCR primers discriminate for and against certain sequences, and that the distribution of rRNA gene amplification products is markedly different from that in the original DNA extract (and target community) [7-15]. It is also known that rRNA gene libraries of typical size (dozens to hundreds of clones) overlap little in their species lists, and that the multiple PCR primer approach appears to detect greater protistan diversity than the use of a single primer set [16,17]. What is not known is whether different clone libraries made from a single DNA source recover species from the same diversity pool, or from a smaller, PCR primers-specific pool of species uniquely amplifiable with these primers. In the former scenario, sequencing such clone libraries to saturation would result in the same species lists (albeit with different species frequency distributions), which would be a faithful representation of the target community composition. This possibility is interesting because massive sequencing is quickly becoming practical with the advance of high-throughput pyrosequencing technology [18-20]. An alternative scenario is that the PCR primer biases are so powerful that complete coverage of sample diversity is impossible using any of the developed general eukaryotic primers, a situation that needs to be considered when assessing protistan richness in a sample, community, or biosphere. Here we address this possibility by analyzing 4 reported 18S rRNA gene clone libraries [16,21] obtained by applying 4 different general eukaryotic primer sets to a single extract of genomic DNA from a stratified water column in the Cariaco Basin off the coast of Venezuela. We apply a combined, multifaceted statistical approach that we developed to estimate microbial richness on the basis of a small sample of this richness [22,23], and we compare the pools of diversity recoverable with each of the four single PCR primer sets to the diversity recoverable from the pooled data.

## Results and discussion

Several aspects of the rRNA approach are widely recognized as biased, most notably the PCR primer bias leading to preferential amplification of some, but not the other, gene sequences [7-15]. The degree of selectivity is not known, and cannot be readily assessed by simply comparing the composition of clone libraries obtained using different primer sets. This is because protistan communities appear to be very diverse and rich in species, and no study has even come close to sequencing rRNA gene clone libraries to saturation. Each reported inventory is therefore only a subset of the target community's complete species list.

While the low overlap between inventories obtained using different primers suggests a strong primer bias [16], it is also possible that it is due to significant undersampling. Considering that there may be hundreds of protistan species in a typical aquatic sample [23], it has been difficult to differentiate between the two explanations. Here we extend a statistical strategy we developed earlier [22,23] to resolve this problem. The test data are four previously published inventories of protistan species, which were obtained by applying four different eukaryotic primer sets to the same source of community genomic DNA. We statistically estimate the sizes of the four diversity pools as they appear from the four individual inventories, and compare those with the fifth estimate obtained from the pooled data. Our logic is simple: if the only difference among these five inventories (four individual and one pooled) is the species frequency distribution, such that what is detected in one library is also in principle detectable in the other library, then all five estimates of total protistan richness should converge on a single value. If on the other hand these estimates are statistically different, it will mean that PCR primer biases are so substantial that some species' DNA is simply not amplified, and such species will be practically undetectable, with some – but not the other – primer sets. In this case, the pooled data should produce an estimate of total richness significantly exceeding any estimate obtained from individual clone libraries.

**Figure 1:**
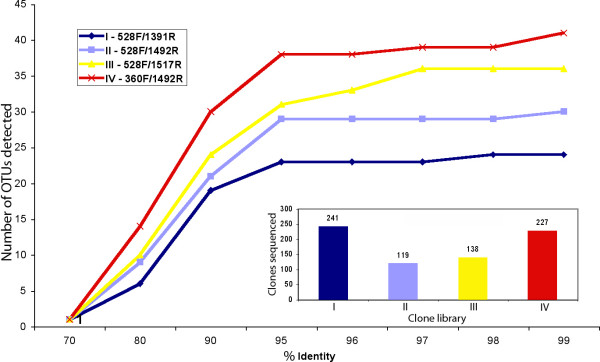
**Observed numbers of OTUs in clone libraries obtained using four different PCR primer sets, as a function of % 18S rRNA gene sequence identity**.

The test clone libraries were obtained as part of our long-term study of protistan diversity in the stratified water column in the Cariaco Basin in the Caribbean, off the coast of Venezuela [16,21]. These studies used a single water sample which served as a source of genomic DNA. This DNA was amplified using separate PCR reactions employing four different eukaryotic primer sets. Here we aligned the overlapping portions of all the sequenced rRNA gene fragments, calculated the all-to-all identity values, and clustered the sequences into operational taxonomic units (OTUs) at different levels of sequence identity (80, 90, 95, 96, 97, 98, and 99%; below 80% the sequence diversity collapsed into a single OTU). The numbers of OTUs per library and PCR primer sets are given in Figure 1. We note that the overlap of OTU lists between the clone libraries was very small: e.g., at 99% sequence identity, no single OTU was shared among all four libraries; between 0 and 3 OTUs were shared among any given 3 libraries; and between 0 and 5 OTUs between pairs of libraries (data not shown).

The statistical analysis proceeded in three stages. First, we estimated total OTU richness (observed + unobserved) separately for each primer set, plus pooled, at each % similarity cutoff. For comparison we computed both parametric and nonparametric estimates of total richness; the former are probably more reliable for high-diversity microbial data, while the latter are typically biased downward [22] in this setting, but the final results from both methods were in reasonable agreement. For example, for the pooled data at the 99% similarity level, the parametric estimate of total richness (based on a mixture of two exponential abundance distributions) was 319 (standard error 85), and the corresponding nonparametric estimate (based on Chao's ACE1 [24]) was 311 (SE 84).

**Figure 2:**
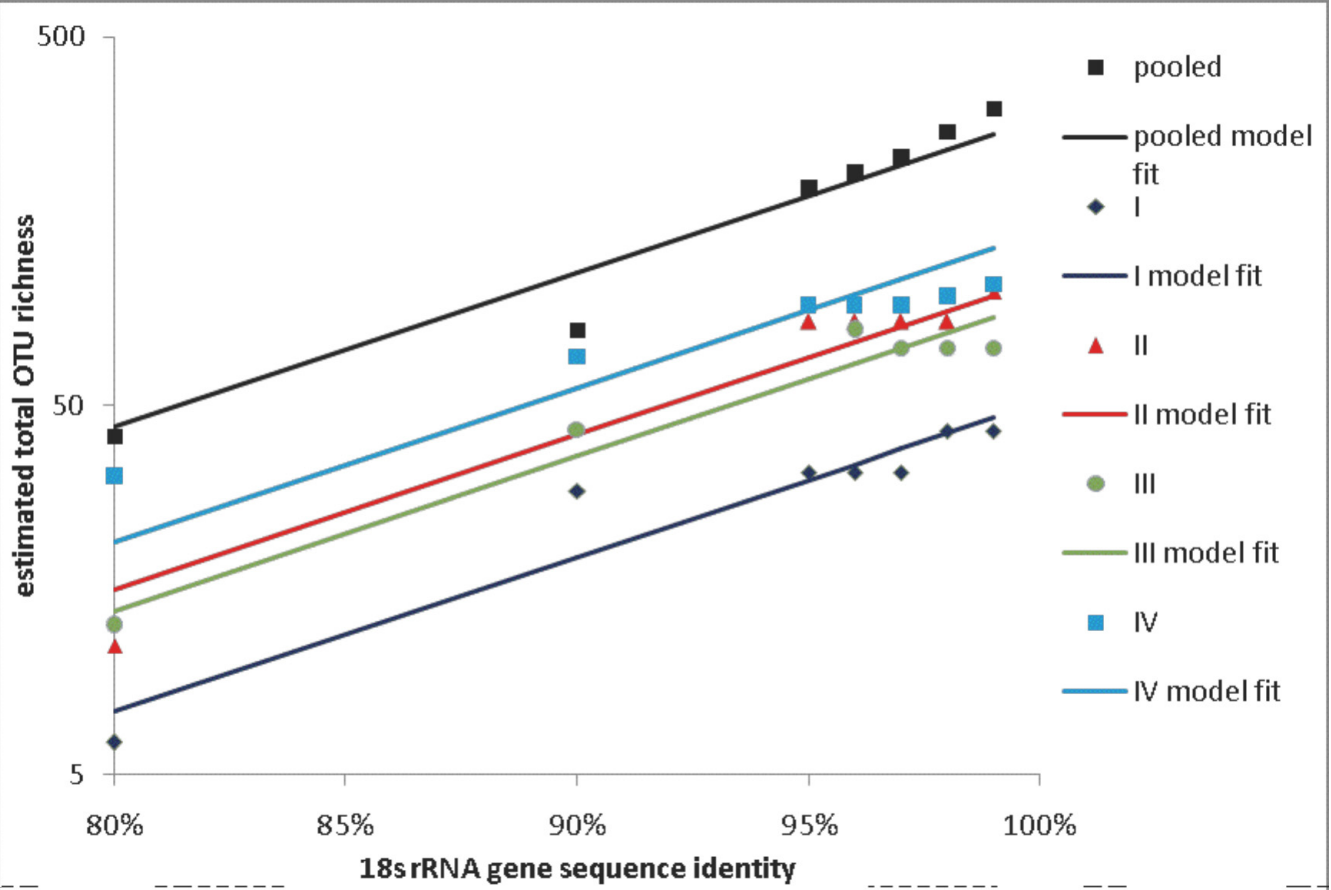
**Total OTU richness as a function of % sequence identity (parametric estimates)**. Estimated total OTU richness (points), and fitted parallel linear regressions (lines) for datasets I, II, III, IV and pooled, as a function of % sequence identity, based on parametric total richness estimates.

Second, we developed a single, combined model for the total OTU richness estimates from all primer sets, plus pooled, at all % similarity levels. We know from substantial empirical experience (Bunge and Woodard, 2008, in preparation) that total OTU richness increases exponentially as a function of % similarity cutoff, or equivalently that log(total richness) increases linearly as a function of % similarity. We therefore introduce a linear (regression) function for each of the four samples, plus pooled, i.e., for each sample we represent the (estimated) total OTU richness as a linear function of % similarity. For large samples this linear increase can be characterized with considerable statistical refinement, and model is clear and consistent across a range of (large) datasets. Here our samples are limited in size (241, 119, 138 and 227, respectively by primer set, and 725 for the pooled sample), which leads not only to statistical sampling variation around the regression line, but also to non-statistical error. The latter is due to the fact that in some cases a 1% increase in the similarity (clustering) level for a small sample did not change the collection of OTUs (clusters), and consequently the corresponding total richness estimates were identical at both similarity levels. We examined a range of statistical approaches (discussed in detail in *Methods*, below) to correct for both sources of error, concluding finally that the model displayed in Figure 2 represents the best analysis of these data for the purpose of distinguishing between the primer sets.

**Figure 3:**
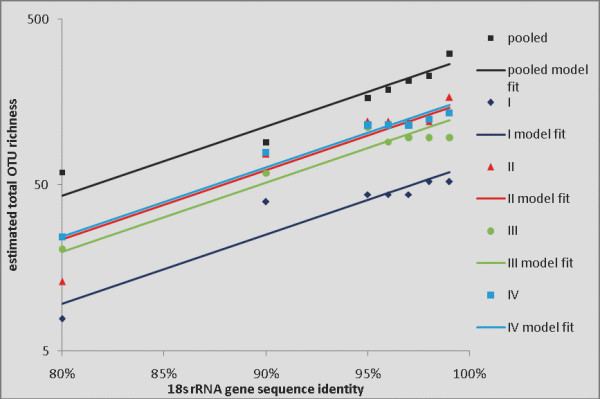
**Total OTU richness as a function of % sequence identity (nonparametric estimates)**. Estimated total OTU richness (points), and fitted parallel linear regressions (lines) for datasets I, II, III, IV and pooled, as a function of % sequence identity, based on nonparametric total richness estimates.

Figure 2 shows the parametric estimates of total OTU richness at each % similarity level, for each of the four primer sets plus the pooled data. There is one linear function for each primer set, plus the pooled data, for a total of five linear functions (regression lines). The lines are parallel (equal slopes); this is supported by statistical testing and empirical experience, and is reasonable for the data considered here. The fit is good (overall R^2 ^= 95.7%) but not perfect (as can be seen from the graph), primarily because of the non-statistical errors described above, which show up as flat spots (unchanged total OTU richness estimates across two or more % similarity levels) in the plots. We concluded that forcing statistical adaptation to this behaviour constituted over-fitting to local artifacts of the data, whereas the parallel linear models represented a good structural summary in this case (see *Methods*). The analogous results for the nonparametric richness estimates are shown in Figure 3. Here the fit is comparable (R^2 ^= 94.4%), but the downward bias of these estimates tends to compress the gaps between the lines.

**Figure 4:**
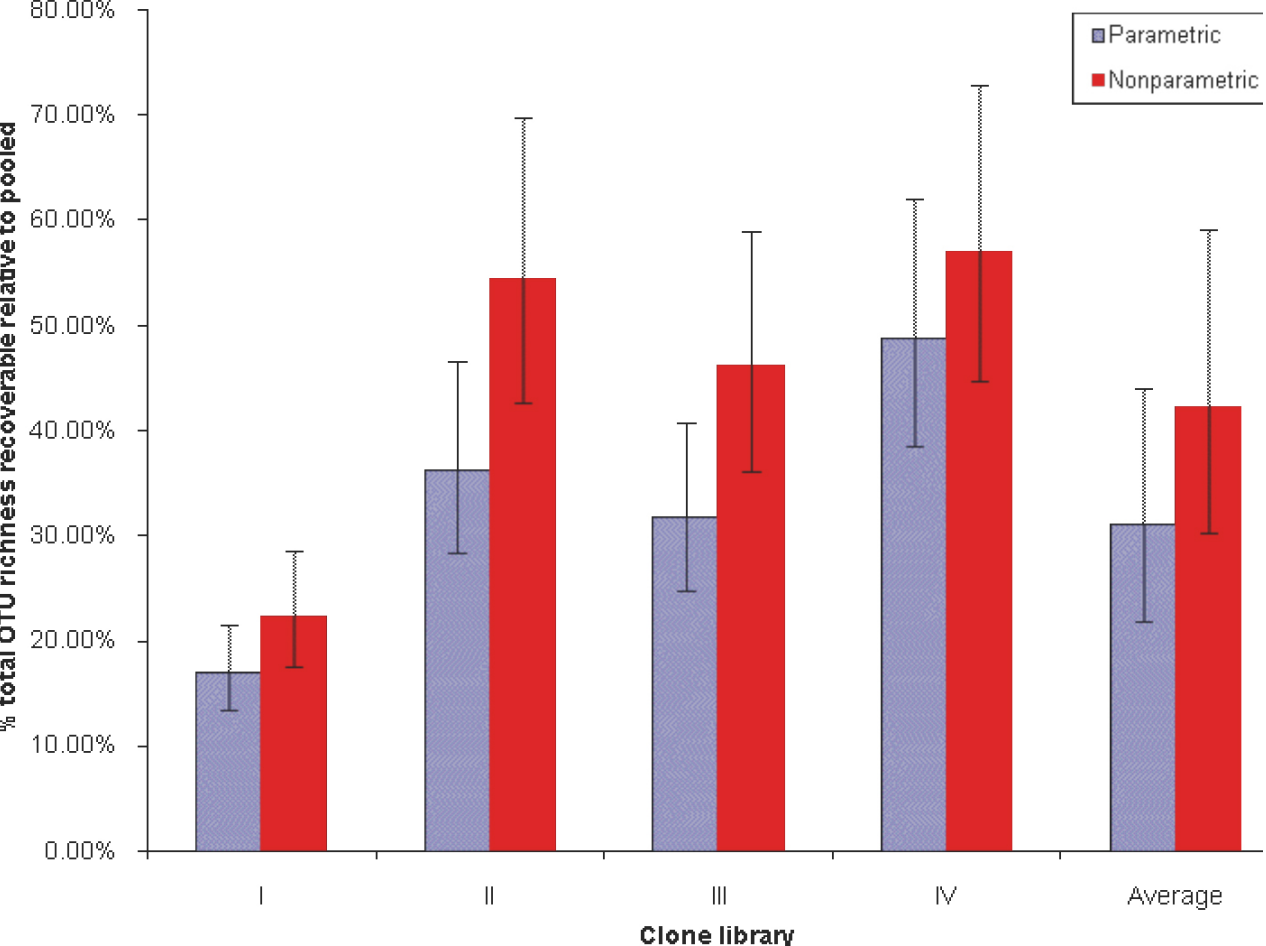
**Estimated total richness recoverable using primer sets I, II, III, and IV, and their average, expressed as % of richness recoverable by pooling data from all four primer sets, with 95% confidence intervals, according to parametric and nonparametric models**.

Third, we considered the differences between the total richness recoverable using each of the four primer sets vs. that recoverable using the pooled data. In terms of the linear model, this is expressed by the differences in the elevations (intercepts) of the parallel (regression) lines. Using the linear (regression) analysis, we estimated these differences, converted them back to the original scale (numbers of OTUs), and calculated confidence intervals for the differences. These intervals represent plausible ranges for the total OTU richness in principle recoverable by each of the four primer sets, expressed as a proportion (percentage) of the total richness recoverable by pooling the data from all four. The results are shown in Figure 4. Note that the percentages are generally higher for the analyses based on nonparametric richness estimates, reflecting the compression of the lines mentioned above. However, the 95% confidence intervals overlap for each primer set, indicating reasonably good agreement of the analyses. The most optimistic possible conclusion from Figure 4 comes from considering the upper confidence bound for the nonparametric analysis of primer set IV: about 73% of the total richness recoverable by the pooled approach may be recoverable using primer set IV alone.

To overcome some of the statistical uncertainties inherent in analyzing all four primer sets vs. the pooled data, we also aggregated the four separate datasets and compared this aggregate to the pooled data. (Essentially this amounts to averaging the four individual primer sets' results and comparing this average to the pooled results). The results are shown in Figure 4. Again the nonparametric version gives higher results, but the confidence intervals overlap considerably. The most optimistic interpretation of Figure 4 is that, on average, the four primer sets can be expected to recover at most 60% of the total microbial diversity recoverable using the pooled approach.

The above statistical analyses showed that estimates of the protistan richness of the sample based on single PCR primer data sets do not significantly differ, and varied between 43 (SE 17) and 107 (SE 34) species (defined as OTUs grouping sequences that share at least 99% identity) (Figure 2). These analyses also showed that once the four clone libraries' data are pooled, and the new species frequency distribution is modeled, the estimate of the sample's richness grows to 319 (SE 85) (Figure 2). By modeling all of the estimates across all % similarity levels (hence taxonomic rank), we obtained sufficient statistical precision to conclude that each of the PCR-specific primer sets recovers a specific set of species, does not recover other species, and cannot in principle detect all the species in the sample. Each clone library would thus undersample the community even if sequenced to saturation, and the degree of such undersampling varies among the primer sets from 83% (17% recoverable) to 51% (49% recoverable). This means that the pooled data set is richer than individual libraries not because it is a larger collection of sequences but because it is less biased. One practical implication of this is that the microbial richness of a sample is better assessed by two clone libraries (created using different PCR primers) sequenced with X effort each rather than by one clone library sequenced with 2X effort. Increasing the diversity of the primers used on each DNA extract will lead to a more complete inventory of the extract, whereas even an unlimited amount of sequencing applied to a single clone library will only recover a portion of the DNA extract's richness.

An *in silico *investigation of primer specificity points to the importance of primer mismatch in determining the overall recovery. For example, the 528F primer set has at least one mismatch with 39% of 18S rRNA gene sequences in SILVA 18S rRNA gene sequence database, and at least two mismatches with 27% of such sequences. The figures for the 1391R primer are 30% and and 25%, respectively. Assuming that any mismatch prevents an efficient primer binding, and the overall efficiency is the product of individual primer efficiencies, then the 528F/1391R PCR primer set would only amplify 61% * 70% = 43% of SILVA sequences, explaining one half of what our analyses predict this primer set will miss in environmental studies.

We were surprised to see that the same holds at other levels of OTU grouping. It is presently impossible to determine whether a specific value of 18S rRNA gene sequence similarity could point to the organism's position within the α-taxonomy hierarchy. In other words, there is no clear correspondence between the degree of molecular divergence of the OTU and its taxonomic rank. However, since identity above 98–99% most likely indicates a very close relationship, and at 70% the protistan diversity addressed here collapses into one OTU, values in between must cover life forms differing at kingdom-, class-, family, and genus levels. Interestingly, at 80% gene sequence identity, the lowest threshold tested at which the sequences in question fell into more than one OTU, the pooled data still appeared qualitatively richer than individual clone libraries. This means that the single PCR primer approach is not only unlikely to recover all species in a sample, but it misses a substantial number of higher taxa as well.

We note that some PCR primer sets appear to be better than others in recovering target diversity. Both parametric and nonparametric modeling suggest that, out of the four combinations tested, the primer set IV (360F/1492R) recovers the most, and the primer set I (528F/1391R) the least part of the sample's richness (Figure 4). The use of multiple sets to amplify the sample's rRNA gene is clearly advantageous because this minimizes the PCR bias. Because the latter may only be reduced but not completely eliminated, the multiple PCR primer approach would still sample sequences from a portion of community diversity. Combined with high throughput sequencing technologies, this approach may detect all *recoverable *taxa, but still miss species that escape amplification with the given PCR primer sets. We note that the degree of biases we estimated is characteristic of the target (anaerobic) communities. These likely comprise less known species, increasing the probability of primer mismatches. It is possible that the biases in question are less pronounced for aerobic (*e.g.*, water column) protists.

## Conclusion

We demonstrated that standard rRNA inventories of protistan diversity, which typically employ a single PCR primer set to amplify protistan rRNA genes, are not samples from the entire community, but only from a fraction thereof. This seems to be the case at all taxonomic levels, from species to the highest taxonomic ranks. Increasing sequencing effort alone is unlikely to increase this fraction as it is grounded in PCR primer selectivity. Here we advocate coupling an increase in sequence coverage with the use of multiple PCR primer sets, because four such sets used here allow access in principle to larger diversity than a single set. The pooled data predicts that there were 319 species in the test sample. This estimate, while significantly larger than those obtained using individual clone libraries, may still be an underestimate because the use of multiple PCR primers is likely to minimize – rather then eliminate – the sampling biases.

## Methods

### Sequence data

The rRNA survey data used here were obtained from [16,21]. These studies used the same DNA extract from a single 2.3 L sample collected just below the oxic/anoxic interface at a water depth of 340 m in the stratified water column of the Cariaco Basin, off the coast of Venezuela. DNA was extracted as described in [21], followed by PCR-aided amplification of ≈ 1,000- to 1,300-bp fragments of the 18S rRNA gene using four different primer sets: (Library I) E528F 5'-CGGTAATTCCAGCTCC-3' [25]-Univ1391RE 5'-GGGCGGTGTGTACAARGRG-3' [26], (Library II) E528F-Univ1492RE 5'-ACCTTGTTACGRCTT-3' [27], (Library III) Euk A 5'-AACCTGGTTGATCCTGCCAGT-3'-Euk B 5'-TGATCCTTCTGCAGGTTCACCTAC-3' [28] followed by a nested reaction with E528F-Univ1517 5'-ACGGCTACCTTGTTACGAACTT-3' [29], and (Library IV) Euk A-Euk B followed by a nested reaction with 360FE 5'-CGGAGARGGMGCMTGAGA-3' [28]-U1492R. The PCR protocol employed HotStart Taq DNA polymerase (QIAGEN, Valencia, CA) in all cases. The PCR products were cloned, separately for each primer set, commercially sequenced, and the inventories were checked for chimeric sequences using the Check_Chimera command of the Ribosomal Database Project (RDP) [30], as well as neighbor-joining trees with partial sequences (partial treeing analyses [31]). The 18S rRNA gene sequences were grouped into OTUs based on 99, 98, 97, 96, 95, 90, 80, 70, 60, and 50% sequence similarity cut off values. This was achieved by first making all possible pairwise sequence alignments using ClustalW at default settings [32] and calculating percent sequence similarities, followed by clustering of the sequences into OTUs using the mean unweighted-pair group method using average linkages as implemented in the OC clustering program . The OTU grouping was checked manually to verify that all OTUs were assembled at the cutoff level desired.

### Statistical Analysis

The statistical analyses operated on 35 datasets ((four primer sets plus pooled data) *(% similarity levels 80, 90, 95, 96, 97, 98, 99) = 5*7 = 35), and proceeded in three stages. First, we estimated the total OTU richness (observed + unobserved) based on each dataset separately. This can be done using two main families of methods, parametric and nonparametric. The former is probably more reliable for highly diverse microbial data [22], while the latter tends to be biased downward in such cases, but we carried out our complete study using both methods. Here the nonparametric total richness estimates were generally lower than the parametric estimates, and slightly more regular in terms of their variation across primer sets and % similarity levels.

**Figure 5:**
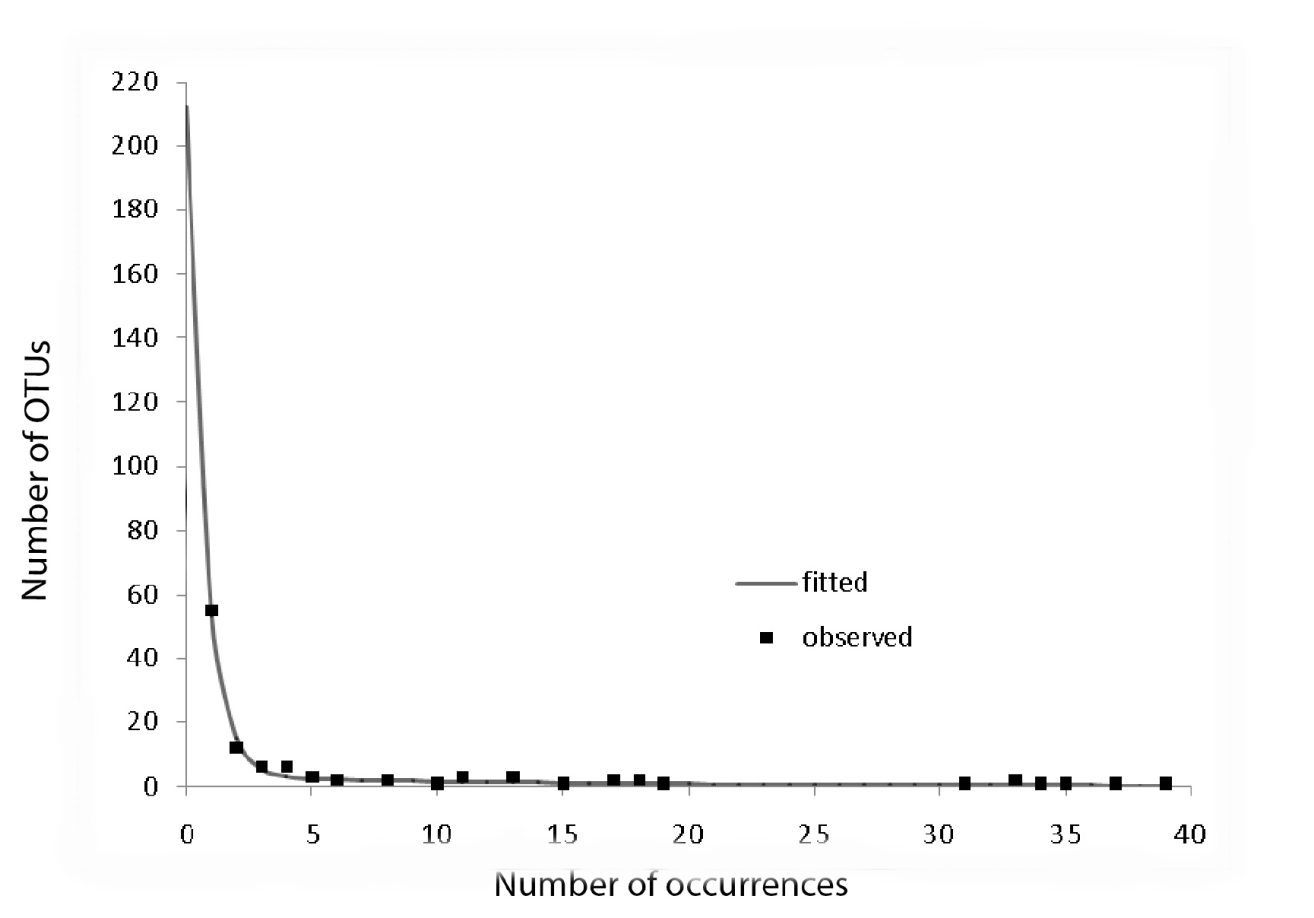
**Parametric model fitted to frequency-count data**. Fit of mixture-of-two-exponentials OTU abundance model (curve) to observed frequency-count data (points), with projection to zero frequency (unobserved OTUs), for pooled data at 99% sequence similarity level (data point at (133,1) omitted for clarity).

To apply the parametric method to an individual dataset, we fit a (parametric) curve to the observed frequency-count (abundance) data by maximum likelihood, and project this curve downward to zero, which yields an estimate of the number of unobserved OTUs, and hence the total number of OTUs (observed + unobserved), along with other important statistics such as standard errors, goodness-of-fit assessments, etc. For example, Figure 5 shows the parametric model fit for the pooled data at 99%: in this case the estimated number of unobserved OTUs was 212 and the estimated total richness was 107 + 212 = 319 (SE 85); the nonparametric estimate ACE1 was 311 (SE 84) (data not shown). We computed both total richness estimates for each of the 35 datasets.

Second, we fit a joint model to all 35 data points simultaneously, using the following logic. We know from empirical experience (Bunge and Woodard, 2008, in preparation) that the total number of OTUs from a given sample (here, the data derived from a particular primer set, or the pooled data) increases exponentially as a function of the % similarity cutoff. Equivalently, the log of the total richness is a linear function of % similarity, i.e.,

log(total richness) ≈ constant + (slope coefficient)*(% similarity cutoff).

**Figure 6:**
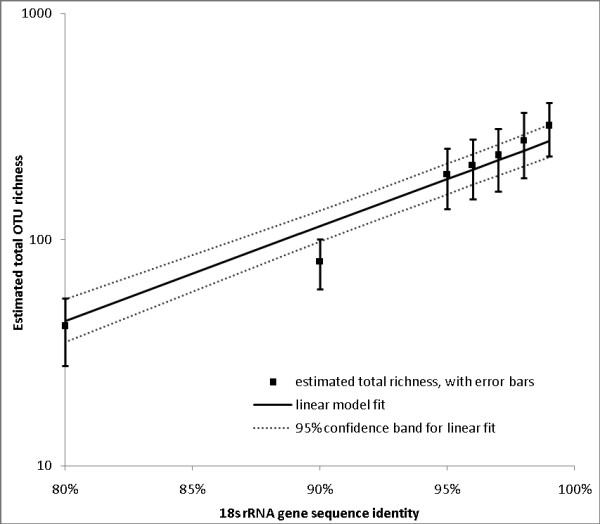
**Total OTU richness based on pooled sample (parametric estimates)**. Estimated total OTU richness as a function of % similarity level, showing original estimates with +/- 1.96 standard error bars, and fitted linear model with +/- 1.96 standard error (95% confidence) band.

This is the main structural relationship. (We note that the total richness estimates derived from a single sample at different % similarity levels are statistically dependent, since they are obtained by re-clustering the same sample; this dependence can be detected and modelled in large samples but not for datasets of the sizes considered here.) The structural relationship allows us to "smooth" the total richness estimates from a given sample across % similarity levels, so that they "borrow strength" from one another, leading to lower overall statistical error for a given sample. Figure 6 shows the linear model fit to the parametric total OTU richness estimates for the pooled data (725) sequences. The original total OTU richness estimates are shown as points, along with 95% error bars, while the total OTU richness estimates obtained by smoothing linearly across % similarity levels is shown by the solid line, along with dashed 95% confidence bands.

**Figure 7:**
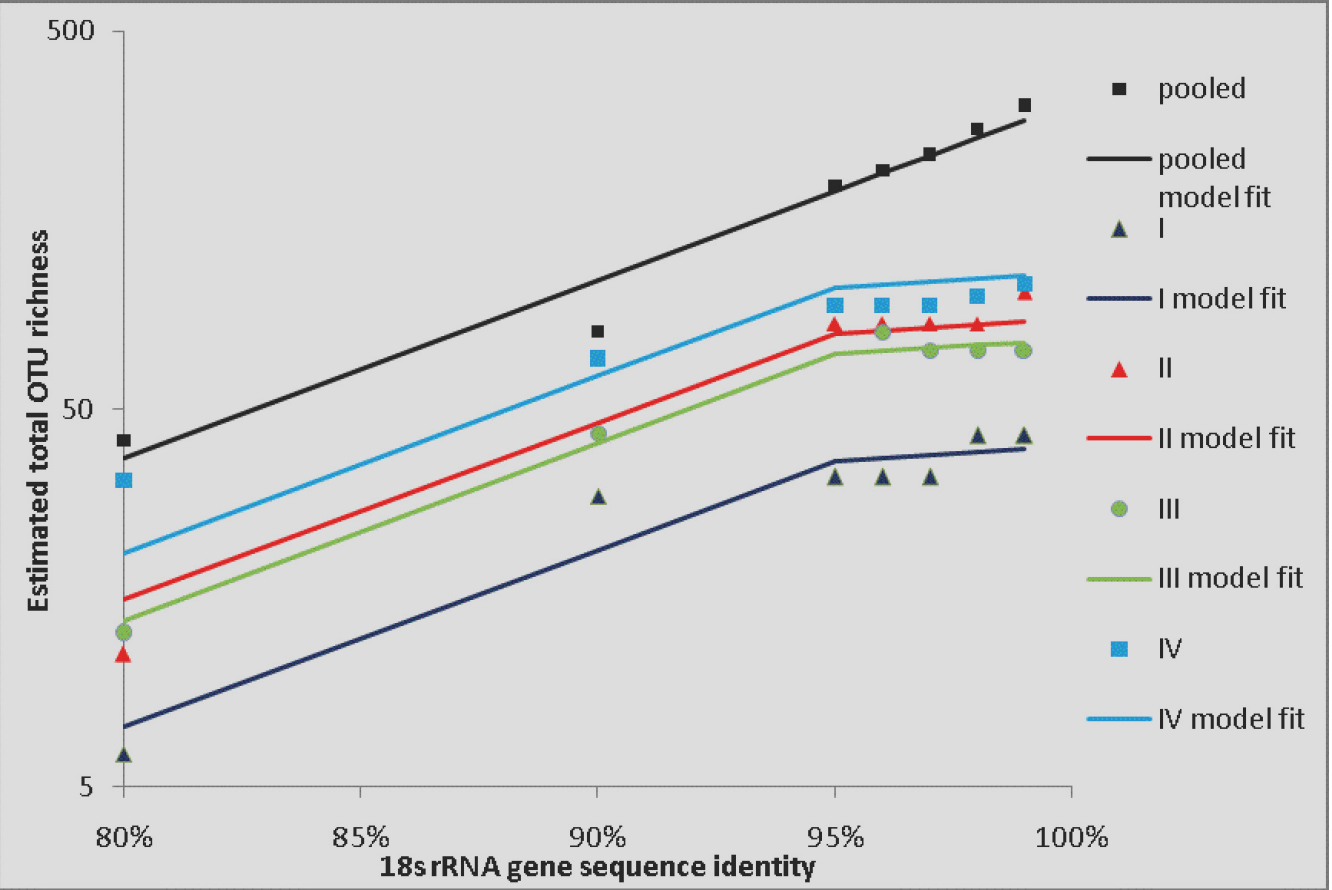
**Total OTU richness as a function of % sequence identity (parametric estimates), changepoint model**. Estimated total OTU richness (points), and fitted regression functions for datasets I, II, III, IV (changepoint model) and pooled (linear model), as a function of % sequence identity, based on parametric richness estimates.

We fit the linear model to each of the five datasets (4 primer sets + pooled), assuming parallel lines (equal slopes); statistical tests showed no difference in the slopes (results not shown). The results are shown in Figure 2 (for simplicity the error bars shown in Figure 6 are not shown in Figure 2). The fit is good (R^2 ^= 95.7%) but not perfect, owing to the fact that the total numbers of sequences processed using the four primer sets were not large (241, 119, 138 and 227, respectively). This led to a combination of statistical and non-statistical errors. The statistical error is reasonably well accounted for by the parallel-lines (regression) model. The non-statistical error arises from the fact that, due to the numbers of available sequences, for some datasets a 1% increment in the % similarity level of clustering did not produce any change in the collection of clusters (OTUs). For example, the OTU clusters produced by the primer set I data were identical at levels 95, 96 and 97% (this was also true for primer sets II and IV). Therefore the estimates of total richness based on sample I are identical at levels 95, 96, and 97% (since they are based on the same data); this is shown by "flat spots" in Figure 2 (and the same holds for datasets II and IV). It is possible to introduce a changepoint into the linear regression line to account for this, so that the line slopes less steeply (flattens out) to the right of a given % similarity value such as 95%. Figure 7 shows such a model, with the changepoint set at 95% for the individual primer set samples, but with no changepoint for the pooled sample. This model is plausible *prima facie*, but given the statistical uncertainty in the total richness estimates (cf. the error bars in Figure 6), and other statistical and empirical considerations, we concluded that the changepoint model overfits local artifacts of the data and distorts the underlying structure, and it also tends to exaggerate the differences between the total richness recoverable via the different primer sets. We therefore adopted the parallel-lines model (Figure 2) as the summary analysis. Figure 3 shows a comparable analysis (to Figure 2) using the nonparametric estimates of total richness. The fit is similar (R^2 ^= 94.4%) but here the downward bias of the richness estimates tends to compress the difference between the four primer sets and the pooled estimates.

Third and finally, we examined the differences between the four primer sets and the pooled data. These differences can be seen in the vertical displacements of the lines in Figure 2 and 3: they have the same slope but different elevations (intercepts). The regression analysis yielded estimates of the elevations, and hence of the differences between them; we then converted these back from the log-scale to the original scale, and calculated Bonferroni-corrected 95% confidence intervals for them. These confidence intervals represent plausible ranges for the total OTU richness in principle recoverable by each of the four primer sets, expressed as a proportion (percentage) of the total richness recoverable by pooling the data from all four. The results are shown in Figure 4. Note that the percentages are generally higher for the analyses based on nonparametric richness estimates, reflecting the vertical-scale compression of the lines mentioned above. However, the 95% confidence intervals overlap for each primer set, indicating reasonably good agreement of the analyses.

To overcome some of the statistical uncertainties inherent in analyzing all four primer sets vs. the pooled data, we also aggregated the four separate datasets and compared this to pooled. Conceptually this amounts to averaging the four individual lines in Figure 2 (or) 3, and comparing the resulting line to the line derived from the pooled data. The results are shown in Figure 4. Again the nonparametric version gives higher results, but the confidence intervals overlap considerably.

Finally we considered potential artifacts due to sample size. It is known that all statistical estimators of total population richness are biased for finite samples. However, the degree and direction of this bias are not known in general, and mathematical analysis of this problem has revealed considerable complexity, which is beyond the scope of our discussion here (Bunge and Barger, 2008; Mao and Lindsay, 2007). In order to assess whether, in the present situation, the differences between the estimated richness based on the individual primer samples, and the estimated richness based on the pooled sample, could be attributed to this bias, we carried out a simulation study as follows.

i. We set the model fitted to the pooled data at the 97% similarity level to be the "true" population distribution. (The abundance distribution here was a mixture of two exponentials, θ_3 _*exp*(-λ/θ_1_)/θ_1 _+ (1-θ_3_)*exp*(-λ/θ_2_)/θ_2_, λ > 0, with θ_1 _= 0.2405, θ_2 _= 3.0954, and θ_3 _= 0.9512.)

ii. We simulated reduced samples from this population, in proportion to the sample sizes for each of the four primer sets in our real data.

iii. For each simulated sample we estimated the total population richness.

iv. We replicated the entire "experiment" 10 times, i.e., we obtained 10 estimates for each of the four reduced sample sizes.

**Figure 8:**
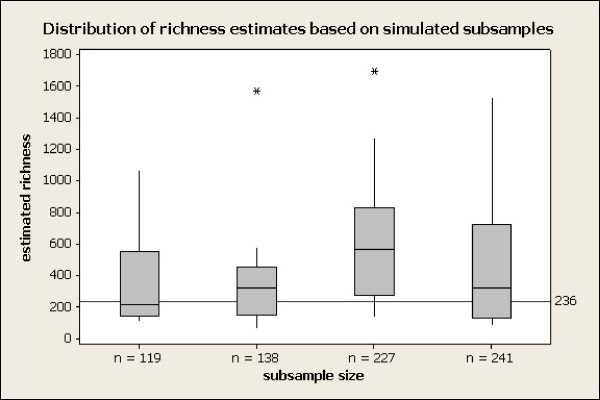
**Distribution of richness estimates based on simulated subsamples**. Distribution of 10 simulated replicate richness estimates, at each of 4 (sub)sample sizes, 97% similarity level, compared to "true" (postulated for this simulation) richness = 236 OTUs. Lower end of box = 1^st ^quartile; central line in box = median; upper end = third quartile.

The results are summarized in Figure 8. For each of the four reduced sample sizes, the range of the 10 simulated richness estimates "covered" or included the "true" (postulated) total richness of 236. (In fact the results displayed a slight positive bias, although assessment of this bias would require mathematical analysis not simulation. More replications would give a slightly better picture but we were constrained by resources; for example, this simulation took 36 hours of computer time.) The standard errors attached to the richness estimates based on the simulated samples were reasonable, and congruent with those obtained from the real data analysis; in particular the occasional large outlier richness estimates were accompanied by correspondingly high SEs (results not shown). This illustrates that, at least under good conditions (when the parametric model applied by the analyst is close to the true, operative population abundance distribution), richness estimators behave well and are not substantially biased downward even for relatively small (~102) samples. Thus we are confident that the reduced richness inferred from the individual primer sets (relative to that inferred from the pooled data) is not an artifact of smaller sample sizes.

## Authors' contributions

SJ, JB, and SSE conceived the study, participated in its design, and drafted the ms. CL performed sequence alignment and clustering the sequence data. JB performed the statistical analysis. TS collected the original sequence data, and SH helped organizing these data for statistical analyses and drafting the ms. SSE coordinated the study. All authors read and contributed to writing the manuscript and approved its final version.
